# Six things to know about the homicides of doctors: a review of 30 years from Italy

**DOI:** 10.1186/s12889-021-11404-5

**Published:** 2021-07-05

**Authors:** Liliana Lorettu, Alessandra M. A. Nivoli, Irma Daga, Paolo Milia, Cristiano Depalmas, Giancarlo Nivoli, Saverio Bellizzi

**Affiliations:** 1 Psychiatric Clinic, Department of Medical, Surgical and Experimental Sciences, University of Sassari, Sassari, Italy; 2 Medical Epidemiologist, Independent Consultant, Geneva, Switzerland

**Keywords:** Homicides of doctors, Stalking, Workplace violence

## Abstract

**Background:**

Healthcare workers have a 16 times greater risk of suffering workplace violence than workers in other sectors and around 50% experience workplace violence in the course of their career. The objective of this study is to explore the characteristics and circumstances of work-related killings of doctors.

**Methods:**

Work-related homicides of doctors over the period 1988–2019 were identified retrospectively through the Italian national statistical agencies. Variables such as perpetrator, motive and location of the crime were obtained through forensic psychiatric work. After classification, the absolute and percent values of the main characteristics of the homicides were calculated.

**Results:**

Over the period considered, 21 doctors were killed in Italy in connection with their professional activity. In 52% (*n* = 11) of cases, the killer was one of the doctor’s patients, in 29% (*n* = 6) of cases it was a patient’s relative, in 19% (*n* = 4) an occasional patient (first consultation). The location of the homicide was a community clinic in 48% (*n* = 10) of cases, the street in 19% (*n* = 4) of cases, the doctor’s home in 14% (*n* = 3), the hospital in 14% (*n* = 3) and the patient’s home in 5% (*n* = 1). In 57% (*n* = 12) of cases the perpetrator was not affected by any mental disorders. The motive for the homicide was revenge in 66.7% (*n* = 14) of cases; in 28.6% (*n* = 6) the revenge was preceded by stalking.

**Conclusions:**

Doctors should be aware that the risk of being killed is not limited to hospital settings and that their patients’ family members might also pose a threat to them.

## Background

Workplace violence is defined as any event that results in harm caused by work-related assaults, threats and abuse, whose impact on the victim may lead to a deterioration in health, safety and well-being [1]. Workplace violence (WPV) is often associated with the type of occupation, with higher incidence among professions involving interactions with many individuals; therefore WPV is a matter of considerable concern for the health sector.

A US report notes that on average, 20 workers are murdered and 18,000 are assaulted each week while at work [2]; similar figures are provided by European reports [3]. Forty-eight per cent of non-fatal workplace violence incidents take place in the healthcare sector [4]. About 50% of healthcare workers experience workplace violence in the course of their career [5, 6]. Healthcare workers have a 16 times greater risk of suffering workplace violence than workers in other sectors [7]. Nurses are more at risk [8, 9] and female workers, both nurses and doctors, are at even higher risk [10]. In a sample of 1826 health professionals, about 11% had suffered physical assault, 5% on more than one occasion, while 64% had received threats or verbal abuse [11]. Saeki et al. [12] report a prevalence of 15%.

Data from the US National Crime Victimization Survey for the period 2005–2009 show a rate of workplace violence of 5.1/1000 for all occupations, 10.1/1000 for physicians and 8.1/1000 for nurses. For mental health workers the violent victimisation rate was 20.5/1000, second only to the rate of law enforcement officers (47.7/1000) [13].

In Belgium, a study on patient-physician aggressions [14] conducted by means of an online questionnaire which was completed by 4930 participants, found that, in the preceding 12 months, 37% had been the victim of aggression: 33% verbal aggression, 30% psychological, 14% physical and 10% sexual. Psychiatric and emergency departments were the settings where violence most commonly occurred.

In Israel, Carmi-Illuz [15] compared the risk of violence between a sample of hospital-based physicians and a sample of community-based physicians, finding a substantially comparable risk.

A particular form of violence, homicide, is infrequent but extremely disturbing.

In 2006, a leading schizophrenia specialist, W. S. Fenton, was killed at his office by one of his patients [16].

In the healthcare professions, homicide is a malicious and intentional event and is a very rare violent circumstance.

We have carried out a retrospective analysis, to explore the main features of all the reported cases of work-related homicides of physicians in Italy over the past 32 years.

## Methods

We relied on the main national statistical databases (ISTAT, EURISPES, EU- RES) [17–19] and on PubMed to trace all cases of work-related homicides of doctors in Italy from 1988 to 2019. The group was completed using the documentation from the forensic psychiatric work of the Psychiatric Clinic of the University of Sassari, Sardinia, Italy.

The Italian Institute of Statistics (ISTAT) provides annual bulletins on homicides and other type of violences (e.g. intimate partner violence) disaggregated by determinants such as Region, gender and age, and type of work. We accessed the annual bulletin-specific databases to obtain te aggregate number of health-worker related homicides [17].

The EURIPSES on the other hand, is a national private agency that operates since 1982 on research in three main fields: social, political and finance. A specific national bulletin is released every year and deals with many aspects of the Italian society, including violence disaggregated by place and type of work [18].

Finally, the EURES represents another national Institute that since 1990 is intended to make research on socio-economic areas with studies at national and sub-national level. The EURES also contains a specific database on homicides and related aspects like relationship between victims and assaulters, motivations, and risks analysis [19].

Based on all the available information from the afore-mentioned database, we first triangulated the information on figures related to homicides of medical doctors under our study period.

Additionally, we searched PubMed for relevant articles. The search terms included: “homicide”; “doctor”; “medical”; “assault”; “aggression”; “nurse”; “health workers”; “hospital”; “health-care”; “kill”; “death”; “Italy”; “murder”; “physician”. Both articles published in English and Italian were considered for this review.

The criteria for selecting reports included the presence of murder or other closely related synonims and the exposures of interest (e.g. being a medical doctor). Epidemiological studies of any health outcome and of any study population as well as of any design, including cross-sectional, case–control and cohort studies, were considered. Two reviewers (LL and SB) evaluated the eligibility of studies. In case of discrepancy a third reviewer (AN) provided arbitration.

The initial search provided 13 non-duplicate records, of which 12 full texts were assessed for eligibility. After exclusion of 5 records that did not meet the pre-established inclusion criteria, 7 studies were retained for qualitative synthesis. Of these 7 studies, 5 combined exposure and outcomes.

While the literature mostly focuses on workplace violence, particularly in hospital settings, and is often primarily centred on psychiatric patients, our study has broadened the scope beyond hospital settings and psychiatric patients to include all cases of work-related doctor homicides.

Specific variables were extracted from each dataset and their value as a percentage of the total was calculated.

The victims’ and the perpetrators’ ages were grouped in 10-year intervals while the geographical location was divided into “Northern Italy” “Central Italy” and “Southern Italy and the Islands”. The method of killing has been classified as “cold weapons” (referring to any weapon that does not involve fire or explosion), “firearms” and “other”, while the locations of the homicide have been classified as “community-based clinics”, “hospitals”, “street”, “perpetrator’s home” and “victim’s home”. In addition to their gender (male/female), the offenders have also been classified according to whether they were psychiatric patients or not. A further distinction concerned the type of patient (“regular patient”, “occasional patient”, or “family member”) and motive (“revenge”, “crime of passion”, “other”).

## Results

From 1988 to 2019, 21 physicians were killed in work-related circumstances in Italy, which means around 0.7 physician killed per year; slightly more than 20,000 overall homicides have been carried out in the same time-period in Italy.

**Fig. 1:**
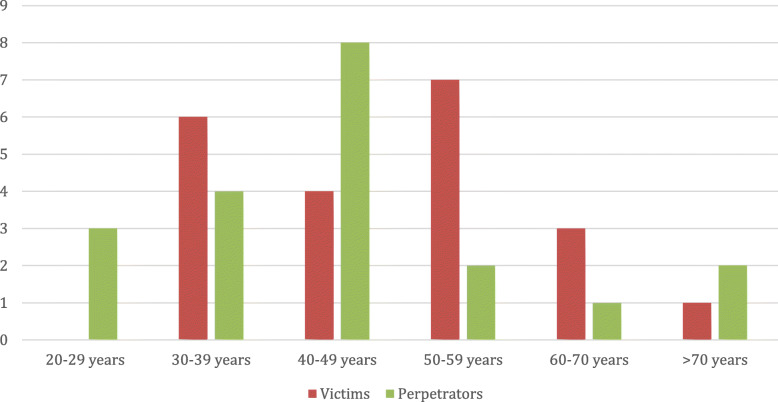
Age distribution of doctors murdered and their perpetrators in Italy, from 1988 to 2019

The victims were more often male (*n* = 15.7%), with a higher concentration (Fig. 1) in age group 50–60 (*n* = 7; 33.3%).

Geographically, the homicides were more prevalent in Southern Italy (South and Islands) with 57.1% of cases (*n* = 12), while only 9.5% were recorded in Central Italy (*n* = 2) and the remaining 33.3% in Northern Italy (*n* = 7).

As to method, 47.6% of the homicides were committed with firearms (*n* = 10) and 42.9% with sharp instruments/knives (*n* = 9). In one case (4.8%) a blunt weapon was used; in another case, the method was poisoning (4.8%).

**Fig. 2:**
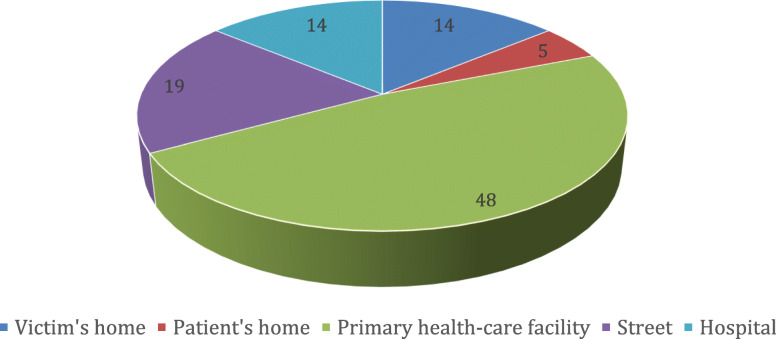
Proportional distribution of murders of doctors by location in Italy, from 1988 to 2019

With regard to location (Fig. 2) about half of the homicides took place outside hospitals, mainly at GPs’ surgeries or out-of-hours primary care services. The most common locations, accounting for 28.6% (*n* = 6) of cases, were GPs’ and out-of-hours surgeries. Next come mental health outpatient clinics, making up 19.0% (*n* = 4) of locations. A further 19.0% of murders occurred in the street (*n* = 4) while 14.3% of cases occurred at the victim’s home (*n* = 3). Three cases took place in hospitals (including adjoining locations such as the car park). One homicide took place at the offender’s home (4.8%).

With regard to the victims’ medical specialties, the most common was psychiatry with an incidence of 28.6% (*n* = 6). This is followed by 4 murders of GPs (19%), 3 of primary care doctors in the out-of-hours service (14.3%) and 2 murders of urologists (9.5%). The remaining cases involved a forensic doctor, an anatomopathologist, a general surgeon, a gynaecologist, an oncologist and a neurosurgeon.

In 52.4% (*n* = 11) of cases, the perpetrators were patients under the victim’s care, while 19.0% (*n* = 4) were occasional patients. The remaining 28.6% (*n* = 6) were family members of the victim’s patients. These latter six cases were all driven by revenge, specifically 4 of the perpetrators who were family members (19.0% of the total) sought revenge for the patient’s death. In 2 out these latter 4 cases, the perpetrators were two fathers seeking revenge for the death of their little girls.

In 66.7% of the cases recorded (*n* = 14), the motive for the murder was revenge without previous threats. In 2 cases, the motive was a worsening of the murderer’s health. A further 2 cases were crimes of passion.. In only one case did the claimed motive originate after the victim had stopped treating the perpetrator. In another case, the motive for the murder is not entirely clear even though the offender, a psychiatric patient on probation, blamed both his victim and all the other doctors at the mental health clinic for the regime he was subjected to (i.e. for being required to report to the Mental Health Centre, MHC, every 3 days to receive treatment). In the last case, the murderer was a medical psychologist who was both a patient and a colleague of the victim, and who took revenge on his colleague for having been subjected to two compulsory mental health hospitalisation orders at the healthcare facility where he had previously been employed.

In 6 cases (28.6%), the motive was revenge preceded by the offence of stalking/threats. Specifically, 2 cases were passion-related: the victims were two women doctors who had long been subjected to threats and stalking by their patients. In one case the murder was preceded by a caution issued by the police.

However, 23.8% of cases (*n* = 5) are not explained by either passion or revenge. Indeed, 3 murders were committed in the out-of-hours general medical facilities by occasional patients (2 drug addicts and 1 intoxicated person) while 2 other cases took place at MHC outpatient clinics.

Analysis of the data collected also shows that in 14.3% (*n* = 3) of cases the homicide was followed by the offender’s suicide and in 9.5% (*n* = 2) the perpetrator killed more than one person.

**Fig. 3:**
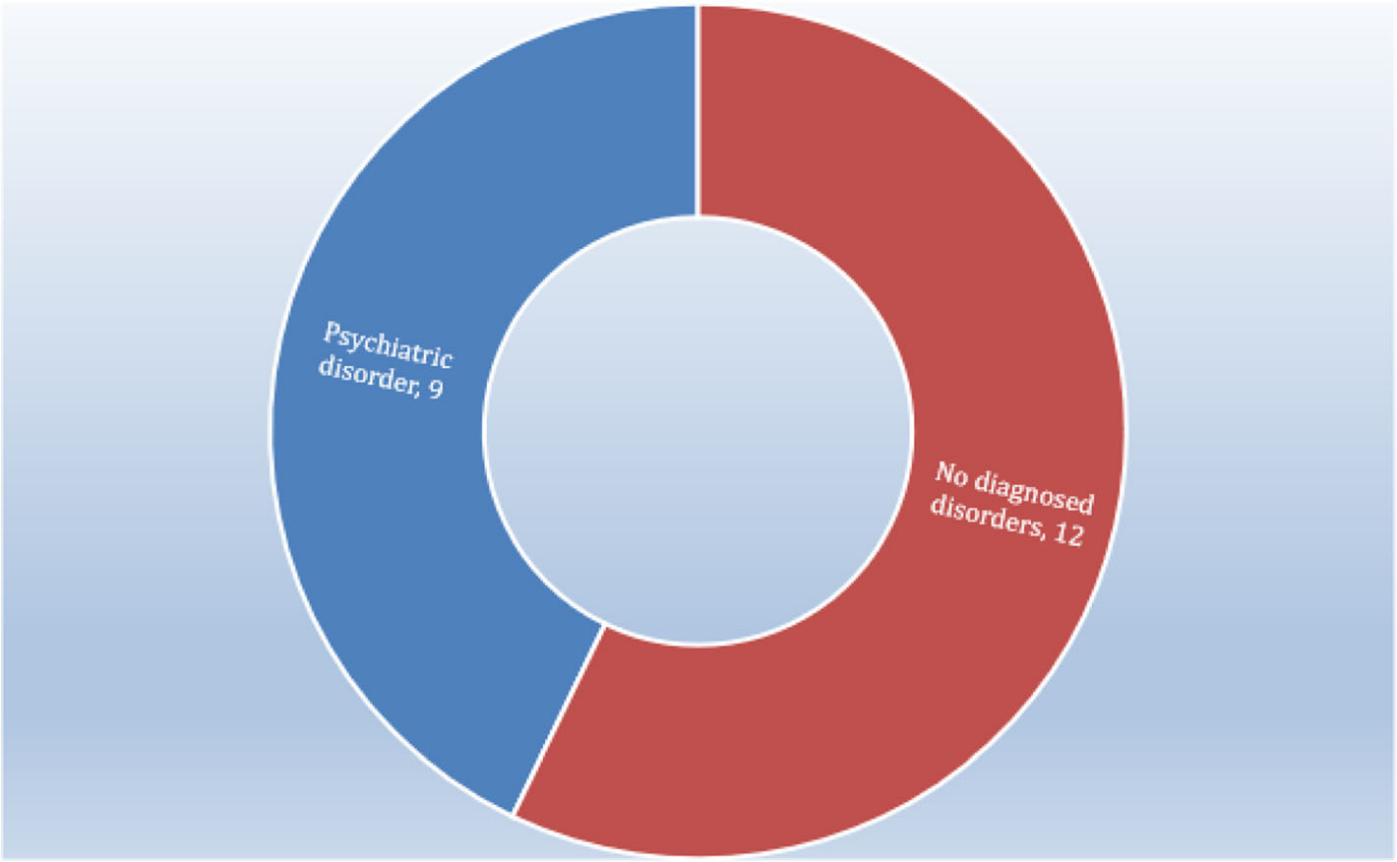
Clinical features of perpetrators of murdering of doctors in Italy, from 1988 to 2019

Lastly, 42.8% (*n* = 9) of the perpetrators had a psychiatric disorder while the remaining 57.1% (*n* = 12) had no diagnosed disorders at the time of the homicides (Fig. 3).

## Discussion

Based on analysis of the data, we would like to highlight that murders on doctors are an extremely rare event; however, some relevant remarks can be made on the characteristics of the phenomenon and possible preventive measures.

Firstly, the literature and statistical data confirm that the medical profession is a dangerous one. In addition to the constant risk of professional liability due to an increasing number of malpractice claims, doctors are exposed to the risk of physical assaults at work and, in extreme and fortunately rare cases, of being killed in connection with their profession.

The US BLS (Bureau of Labour Statistics) (OSHA 4) reported that 69 healthcare workers (HCWs) were killed between 1996 and 2000 [20]. In Italy, in the period between 1988 and 2010, 17 physicians were killed at work [20].

The literature highlights the aggressiveness and violence of patients in hospitals and particularly the risk associated with psychiatric patients [21–25].

We have analysed the cases of physician homicides linked to the doctor-patient professional relationship, both within and outside clinical settings. The sample we examined supports more complex reflections.

The first reflection concerns the location of the homicide: in most cases it was not the hospital. Out of 21 cases, only 3 homicides were committed in the hospital or in nearby places (e.g. car park), while the other murders took place outside the hospital. In particular, a significant number of murders occurred in outpatient settings (community clinics), while still others occurred at the patient’s home, at the doctor’s home or in casual places (on the street). In a particularly striking instance, the patient took a revenge on the doctor: he sent a poison-laced bottle of wine as a Christmas gift to the victim’s home for the Christmas holidays, causing the doctor’s death. This unusual and horrifying case confirms that the homicidal intent may play out beyond healthcare settings and reach the victims elsewhere, including in their own home. The significant number of homicides in places other than hospitals calls into question the limited scope of preventive measures, which so far have focused on hospital settings. Some authors [26, 27] have reported a sharp decline in violent behaviour against healthcare workers after the introduction of specific security measures in hospitals. Similar systems would certainly also be effective in community clinics, where almost 50 homicides have taken place [17]; these outpatient settings as a rule have insufficient security arrangements and would require the implementation of security protocols.

Another observation concerns the type of offender. In our sample, the perpetrators included not only the doctor’s regular patients, but also patients’ family members and occasional (drop-in) patients. With regard to the authors of violent acts in the workplace, Rippon [28] identifies several categories: patients, family members, visitors and co-workers. In Turkey, a study found that 64.5% of the attacks were carried out by the patients’ family members [29].

This data also exposes the limitations of violent behaviour risk assessment and management systems in reducing the risk of violent behaviour by patients [24, 30]. While these tools are certainly useful when dealing with patients registered with healthcare facilities, they may fail to prevent homicide in cases where the perpetrator is a patient’s family member or an occasional/drop-in patient, as these types of individuals cannot be subjected to risk assessment. In the sample analysed, almost half of the perpetrators of homicide were patients’ family members or occasional patients.

The sample reveals a “wide scope of danger” both in terms of location, which extends beyond the hospital setting and may also include the doctor’s or the patient’s home, and in terms of offenders, who may also be patients’ family members.

Some remarks can also be made about the motives for the murder in the sample. In a large number of cases the motive was revenge against the doctor for a claimed error in diagnosis, surgery or treatment causing harm to the patient. In a number of cases the murder did not occur as an escalation of an outburst of violence but was the outcome of planned and premeditated revenge against the doctor. Often the revenge was preceded by threats.

In some cases the murder was preceded by stalking. While the instances of stalking seem to be relatively few, their number may be an underestimated also due to the fact that this behaviour has been recognised as an offence only in recent years. Therefore, it would be appropriate to highlight the role of stalking as a risk factor for homicide, and to raise public awareness of this behaviour to improve its management and prevent its escalation into violent acts. While of course stalking behaviour is not necessarily a precursor of murder, a useful recommendation is to report all stalking behaviours and take specific precautionary measures.

Finally, psychiatric patients warrant specific remarks. Large and Nielssen [30] have analysed homicides by psychiatric patients in psychiatric hospitals and proposed a classification into three patient categories: acute psychiatric patients soon after hospital admission, patients not receiving medication with a history of serious violence, and patients with dementia or intellectual disability, held in low-security inpatient settings in contact with vulnerable patients (victims of the homicide). In this study, the victims were both healthcare workers and fellow patients.

In the sample we analysed, 43% of the perpetrators had a diagnosed psychiatric disorder, while 57% did not. We do not have data on how many offenders with psychiatric disorders were found to be not prosecutable due to mental impairment. A study by Knable [22] describes a sample of mental health workers who were killed by patients in the US. The study highlights the low frequency of these events (about one case per year) and describes the characteristics of victims and perpetrators. The victims were most likely young female caseworkers, with little work experience, killed during unaccompanied visits to residential treatment facilities. The perpetrators were mostly male, diagnosed with schizophrenia, with poor adherence to medication.

The debate about the link between mental illness and violent behaviour is still open and very controversial. In accordance with the literature and in light of the data examined here, we recommend that psychiatric patients be assessed for their risk of violent behaviour, particularly if they have a history of violent behaviour, current substance use and non-compliance with their medication [24, 31–35].

.However, it should also be noted that individuals (patients or family members) who are not mentally ill can also be offenders.

## Conclusions

This paper is significantly limited by the small size of the sample group, which makes it difficult to draw general conclusions.

However, with this caveat, we can highlight six things to know about the homicides of physicians.

1. Physicians may be attacked not only in hospital settings but in other locations too, even at home. Isolated outpatient clinics are at high risk and would benefit from improved surveillance and security systems.
2. The perpetrator is not necessarily a patient, as doctors may be targeted by a family member of the patient seeking revenge.
3. Many cases stem from allegations of medical malpractice against the doctor. Conflict mediation systems should be set up for the parties’ use. In Italy, the Gelli-Bianco Law, on the safety of healthcare and the professional liability of healthcare providers, has introduced compulsory mediation before legal action can be taken.
4. Stalking is a risk factor that should not be underestimated; doctors should be aware that it might precede violent behaviours. Physicians who are victims of stalking should always contact the police.
5. Psychiatric patients, especially those with a history of violent behaviour, substance use and poor adherence to their medication regimen should undergo a specific risk assessment for violent behaviour. However, the sample analysed suggests that the threat is not limited to psychiatric patients.
6. Physicians should receive specific training in effective communication skills and conflict management with patients and their family members, including non-violent communication techniques, listening skills and conflict identification and recognition.

While each case in our sample has unique characteristics, each can be considered a piece of a broader puzzle, which requires more studies in order to move beyond prejudice and silence.

## Data Availability

The datasets generated and/or analysed during the current study are available in the ISTAT (https://www.istat.it/), EURISPES (https://eurispes.eu/) and EURES (https://www.eures.it/) websites. Public access to the databases is open.

## Abbreviations

BLS: Bureau of Labour Statistics
EURES: Istituto Europeo di Ricerche Economiche e Sociali
EURIPSES: Istituto Europeo di Studi Politici Economici e Sociali
GP: General Practitioner
HCW: Healthcare worker
ISTAT: Instituto Italiano di Statistica
MHC: Mental Health Centre
WPV: Workplace violence
